# Effects of the filter microstructure and ambient air condition on the aerodynamic dispersion of sneezing droplets: A multiscale and multiphysics simulation study

**DOI:** 10.1063/5.0053449

**Published:** 2021-06-24

**Authors:** Kyeongeun Lee, Jungtaek Oh, Dongwhan Kim, Jinbok Yoo, Gun Jin Yun, Jooyoun Kim

**Affiliations:** 1 Department of Textiles, Merchandising and Fashion Design, Seoul National University, Seoul 08826, South Korea; 2 Reliability Assessment Center, FITI Testing and Research Institute, Seoul 07791, South Korea; 3 UniAET Co., Ltd., Seoul 08502, South Korea; 4 Department of Aerospace Engineering, Seoul National University, Seoul 08826, South Korea; 5 Institute of Advanced Aerospace Technology, Seoul National University, Seoul 08826, South Korea; 6 Research Institute of Human Ecology, Seoul National University, Seoul 08826, South Korea

## Abstract

Concerns have been ramping up with regard to the propagation of infectious droplets due to the recent COVID-19 pandemic. The effects of filter microstructures and ambient air flows on droplet dispersion by sneezing are investigated by a fully coupled Eulerian–Lagrangian computational modeling with a micro-to-macroscale bridging approach. Materials that are commonly applied to face masks are modeled to generate two different virtual masks with various levels of filtration efficiency, and the leakage percentages through the unsealed nose and cheek areas were set to 11% and 25%, respectively. The droplet propagation distance was simulated with and without mask wearing in still and windy conditions involving head wind, tail wind, and side wind. The results demonstrate that wearing a face mask reduces the transmittance distance of droplets by about 90%–95% depending on the mask type; nonetheless, the droplets can be transmitted to distances of 20–25 cm in the forward direction even with mask-wearing. Thus, a social distance of at least 20 cm between people would help to prevent them from becoming exposed to ejected droplets. This study is significant in that important aspects of mask materials, in this case the porous microstructure-dependent filtration efficiency and permeability under varied ambient flow conditions, were considered for the first time in an evaluation of the barrier performance against droplet transmittance through a multiphase computational fluid dynamics simulation of air-droplet interaction and turbulence flow dynamics.

NOMENCLATURE

$A_{p}$: projected area of the droplet (m^2^)
$A_{s}$: surface area of the droplet (m^2^)
B: spalding mass transfer number (dimensionless)
$c_{p}$: specific heat capacity of the droplet [J/(kg· °C)]
CAD: computer aided design
CDC: centers for disease control and prevention
CFD: computational fluid dynamics
COVID-19: coronavirus disease of 2019
$C_{D}$: drag coefficient of the droplet (dimensionless)
$D_{p}$: mass diffusivity of the liquid droplet (m^2^/s)
$D_{v}$: mass diffusivity of the water vapor (m^2^/s)
$e_{p}$: enthalpy difference that evolves over time (J)
E: total energy (J)
f: body force (N)
FE: filtration efficiency
FVM: finite volume method
$F_{B}$: gravitational body force (N)
$F_{D}$: Stokes drag force (N)
$F_{\mathrm{int}}$: internal force (N/m^3^)
$F_{M}$: added-mass force (N)
$F_{P}$: pressure force (N)
g: gravitational acceleration vector (m/s^2^)
$g^{*}$: mass transfer conductance [kg/(m^2^·s)]
H: height (m)
J: Jacobian of the volume (dimensionless)
k: permeability (m^2^)
KDCA: Korea disease control and prevention agency
KF: Korea filter
KFDA: Korean food and drug administration
KF80: Korea filter mask with 80% of filter efficiency
KF94: Korea filter mask with 94% of filter efficiency
$k_{\mathrm{eff}}$: effective thermal conductivity [W/(m· °C)]
L: length (m)
LR: leakage rate
${\dot{m}}_{p}$: evaporation rate of the droplet (kg/s)
N95: not resistant to oil, filters at least 95% of airborne particles
p: pressure (Pa)
PET: polyethylene terephthalate
PP: polypropylene
RANS: Reynolds averaged Navier–Stokes
${\text{Re}}_{p}$: Reynolds number of the droplet (dimensionless)
S: source [kg/(m^3^·s)]
Sc: Schmidt number (dimensionless)
$S_{h}$: source of total energy [N/(m^2^·s)]
$S^{v}$: momentum source (N/m^3^)
${Sh}_{p}$: Sherwood number (dimensionless)
$t_{f}$: filter thickness, $t_{f}$ (m)
$T_{p}$: temperature of the droplet ( °C)
$u_{p}$: velocity of the droplet (m/s)
U.S.: United States
v: face velocity (m/s)
v: velocity vector (m/s)
$v_{i}$: velocity in the turbulence flow model (m/s)
${\bar{v\;}}_{i}$: average velocity (m/s)
$v_{i}^{\prime }$: fluctuating velocity (m/s)
W: width (m)
WHO: World Health Organization
X: position vector (m)
$Y_{v}$: mass fraction far away from the droplet surface (dimensionless)
$Y_{v,s}$: mass fraction of water vapor in the air near the surface of the droplet (dimensionless)
3D: three dimensions
ΔP: pressure drop (kg/m·s^2^)
*μ*_a_: air dynamic viscosity (kg/m·s)
ρ: density at elapsed time (kg/m^3^)
$\rho_{a}$: air density (kg/ $m^{3}$)
$\rho_{p}$: particle density (kg/ $m^{3}$)
$\rho_{0}$: density at initial time (kg/m^3^)
τ: molecular stress (Pa)
$\tau_{v}$: viscous stress tensor (Pa)
$\tau^{t}$: turbulent stress (Pa)

## I. INTRODUCTION

Human-produced droplets can transmit infectious diseases such as COVID-19 by propagating virus-containing droplets to considerable distances.^1–4^ To reduce the risk of exposure to infectious droplets and aerosols, the routinely recommended procedure is to wear a face mask and to maintain a certain social distance.^5–7^ Such protective actions are relevant because human activities such as sneezing, coughing, and talking account for a significant portion of infection routes, and a face mask represents the first line of protection.^8–14^

The U.S. Centers for Disease Control and Prevention (CDC) mandated the use of face masks on public transportation starting in February of 2021 and recommended a social distance of at least six feet (about two arm lengths, or 2 m) between people.^15^ The World Health Organization (WHO) suggested maintaining a distance of at least three feet between people who cough or sneeze.^16^ In Korea, it is recommended to wear a certified face mask (generally KF80 or KF94) or a “droplet mask.” The regulatory criteria of filtration efficiency (FE) and leakage rate (LR) of KF80 and KF94 masks certified by the Korea Food and Drug Administration (KFDA) are the following: FE ≥ 80% and LR ≤ 25% for KF80; FE ≥ 94% and LR ≤ 11% for KF94. There are no such performance criteria for the droplet mask; instead, it requires water resistance performance to protect against droplets, but not necessarily fine particulates.^17^

Even with those protective measures, it is difficult to establish specific safety guidelines for diverse environmental conditions and various situations.^18^ Thus, it is necessary to understand the aerodynamic movement of droplets in specific situations, implementing different scenarios with various mask performance and ambient air flow conditions. There have been efforts to investigate droplet propagation by simulating human activities. Hui *et al.*^7^ investigated the transmittance distance of droplets through surgical and N95 masks by coughing, and employing a human patient simulator and a laser visualization technique, reported that the surgical mask generated significantly stronger lateral air flows (28 cm of transmittance distance) than N95 mask (15 cm of transmittance distance), due to the higher side leakages for the surgical mask. It should be noted that “surgical mask” in this study refers to a rectangular facepiece without a specific grade of standard certification. In their study, the effects of filter material systems and ambient outdoor air conditions were not considered. Most previous studies^7,9,19–23^ reported that N95-certified masks effectively reduced the number of droplet jets transmitted outside of the mask compared to an uncertified rectangular type of mask.

Tang *et al.*^24^ optically visualized the fluid dynamics by observing air flows around human subjects when they coughed. Their study intended to evaluate the effects of face masks on the fluid dynamics mechanisms by characterizing exhaled airflows produced by coughing. The study was significant in that natural human behavior was observed, adopting the Schlieren optical video method. However, this approach can be employed very limitedly with fewer potential hazards, and it was only a result in a specific condition, not a value reflecting various environmental conditions. More broadly, computational modeling and simulations can be effective approaches by which to investigate the air flow dynamics of infectious droplets with applying various conditions. The versatile capability of multiphase computational fluid dynamics (CFD) simulations allows one to reveal complex droplet interactions, dynamics, and phase changes under various ambient air conditions, which are frequently overlooked in preventive actions and guidelines.^25,26^

In work by Dbouk and Drikakis,^1^ the importance of the concept of social distance was justified, demonstrating that droplet jets produced by coughing could be transmitted through a mask in their fluid dynamics simulation. Employing a multiphase CFD with a fully coupled Eulerian–Lagrangian framework, the droplet dynamics was computed under repetitive cyclic coughing.^27^ Pendar and Páscoa^4^ simulated droplet trajectories by applying a broad range of conditions pertaining to the droplet velocity, size distribution, injection angle of the droplet, the mouth opening area, and certain environmental factors. Despite all of these findings on droplet dynamics in various scenarios, no research has linked the material components of face masks and ambient conditions to the transmittance of droplets.

This study intends to address the effects of mask performances and ambient wind conditions on the droplet propagation distance at different sneezing speeds through a computational simulation. The filtration efficiency and leakage rates of face masks were varied to simulate two different mask configurations and face fits. Two types of virtual face masks with different filtration efficiency rates were constructed by referencing a KF94 certified mask (94% efficiency) and a droplet mask (uncertified for particulate matter). A person's upper body shape and face mask were reverse-engineered via 3D scanning. The filtration efficiency of virtual masks and arbitrary leakage rates through unsealed gaps in the nose and cheek regions were varied in the computation, and their influences on jet propagation outcomes were investigated. A static condition and three wind conditions were applied in the simulation. A fully coupled Eulerian–Lagrangian computational approach was used to analyze the multiphase fluid dynamics of the droplet jet and a turbulent flow. Moreover, the phase change of droplets was considered by means of the two-way coupling of vaporization and a heat transfer.

**FIG. 1.**
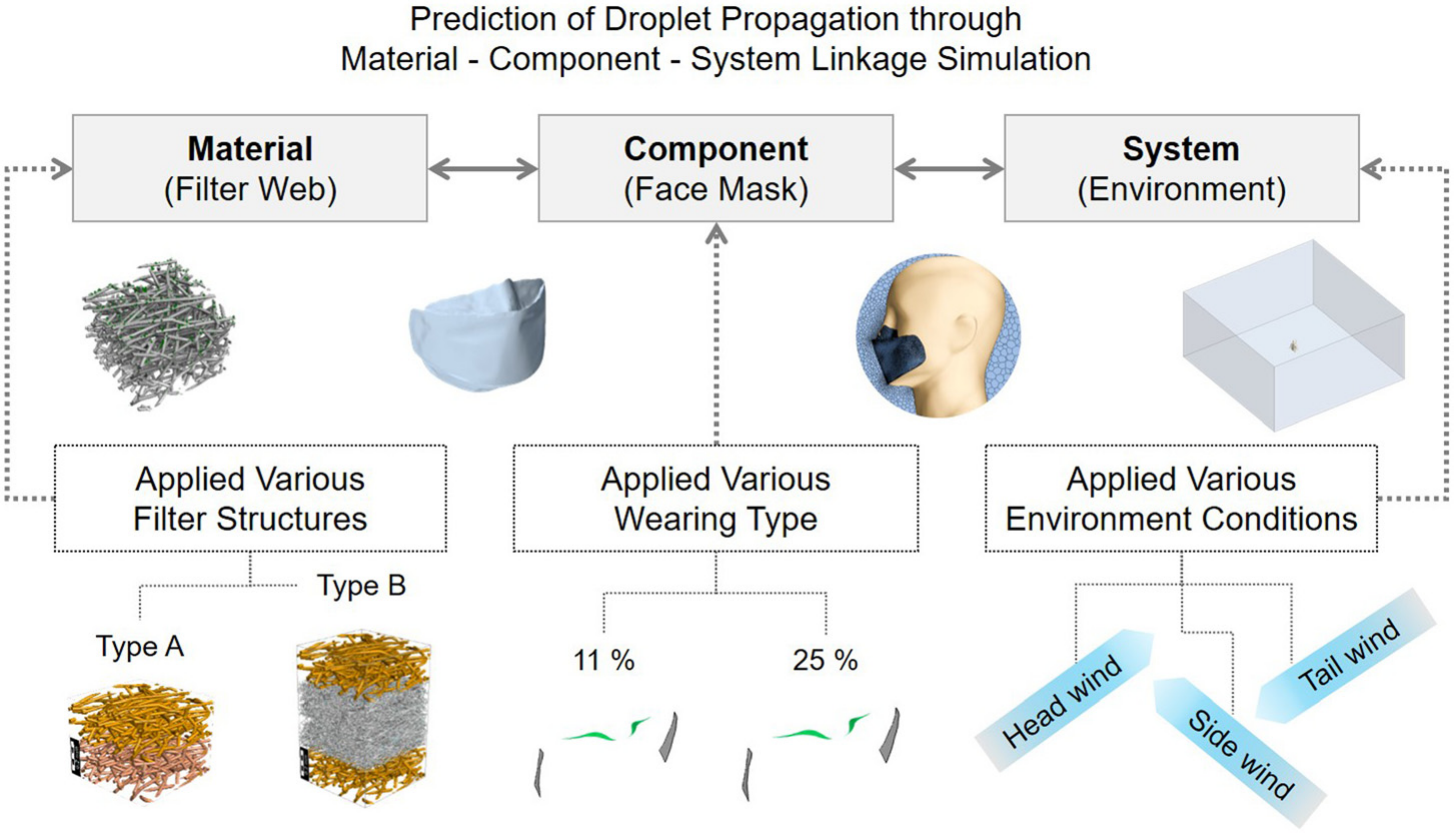
Schematic of research variables, including face mask materials, leakage rate depending on the characteristics in which they are worn, and environmental factors.

This study is significant in which the material aspects of permeability and filtration efficiency, as well as the various ambient conditions, were implemented in droplet transmittance computations. A schematic of the research variables is shown in Fig. 1. Ultimately, the results of this study can serve as informative guidance on proper preventative measures for public health and safety, such as establishing safe social distances and mask specifications for probable exposure scenarios.

## II. SIMULATION AND EXPERIMENTAL METHODOLOGY

### A. Modeling of nonwoven materials for face mask design

The filtration efficiency is defined as the percentage of particles of a certain size that would be filtered by a filter medium. To simulate the actual range of filtration efficiency of virtual masks, spunbond and meltblown webs of the types commonly used for face masks were used as reference nonwoven materials. These nonwoven webs were tested using an automated filter tester (TSI 8130, TSI Inc., USA), exposing an area of 100 cm^2^ to 20 ± 5 mg/m^3^ NaCl particles (mass median diameter of ∼0.6 *μ*m) at a flow rate of 60 L/min. A layer of spunbond web had 9.3% ± 0.7% filtration efficiency, and a layer of meltblown web had 95.0% ± 0.7% filtration efficiency. Two types of virtual face masks webs were constructed, referencing those nonwoven materials, using the FiberGeo^®^ module in the commercial software GeoDict^®^.^28,29^

### B. Coupling of filtration pore microstructures with air flow and droplet dispersion

**FIG. 2.**
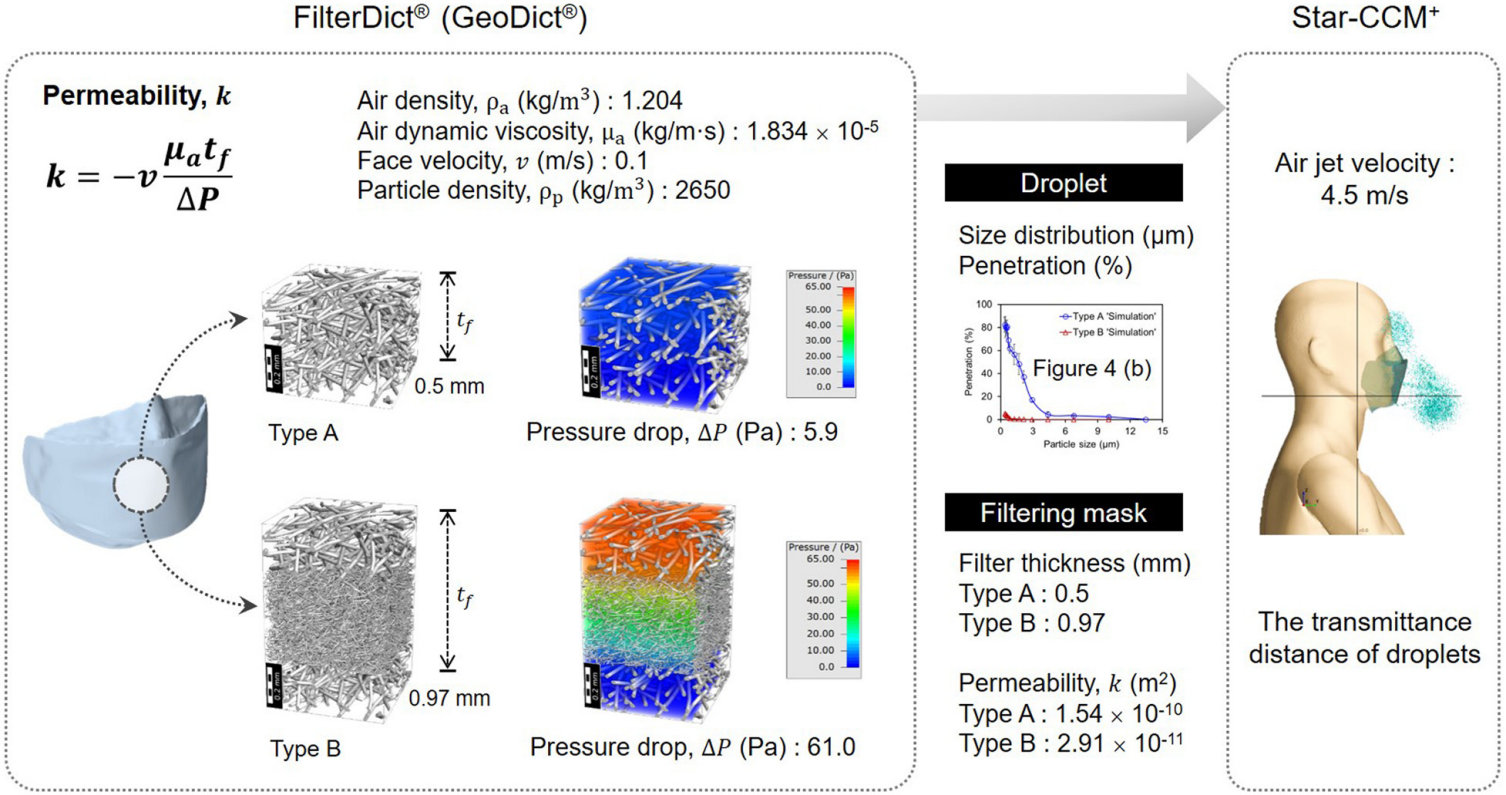
Multiscale modeling approach of the pore microstructure and the aerodynamics dispersion of respiratory droplets.

The interaction between the droplet and porous filter medium was investigated by Dbouk and Drikakis.^27^ They adopted an empirical model originally devised by Bai *et al.*^30^ to describe local interactions between droplets and an impermeable wall. However, the pore microstructures of the filter media were not considered in their study. In the present study, interactions between droplets and a porous filter microstructure are simulated to characterize the droplets through the mask filter and the mask itself, specifically to determine (1) the statistical size distributions of the unsieved droplets, (2) the pressure drop, and (3) the permeability as computed from the pressure drop. The filter-porosity-dependent size distributions and permeability of the mask are sequentially linked to an aerodynamic dispersion simulation of the droplets in Sec. III. Figure 2 shows the proposed multiscale modeling approach for a porous filter microstructure and the aerodynamics simulations of saliva droplets.

**FIG. 3.**
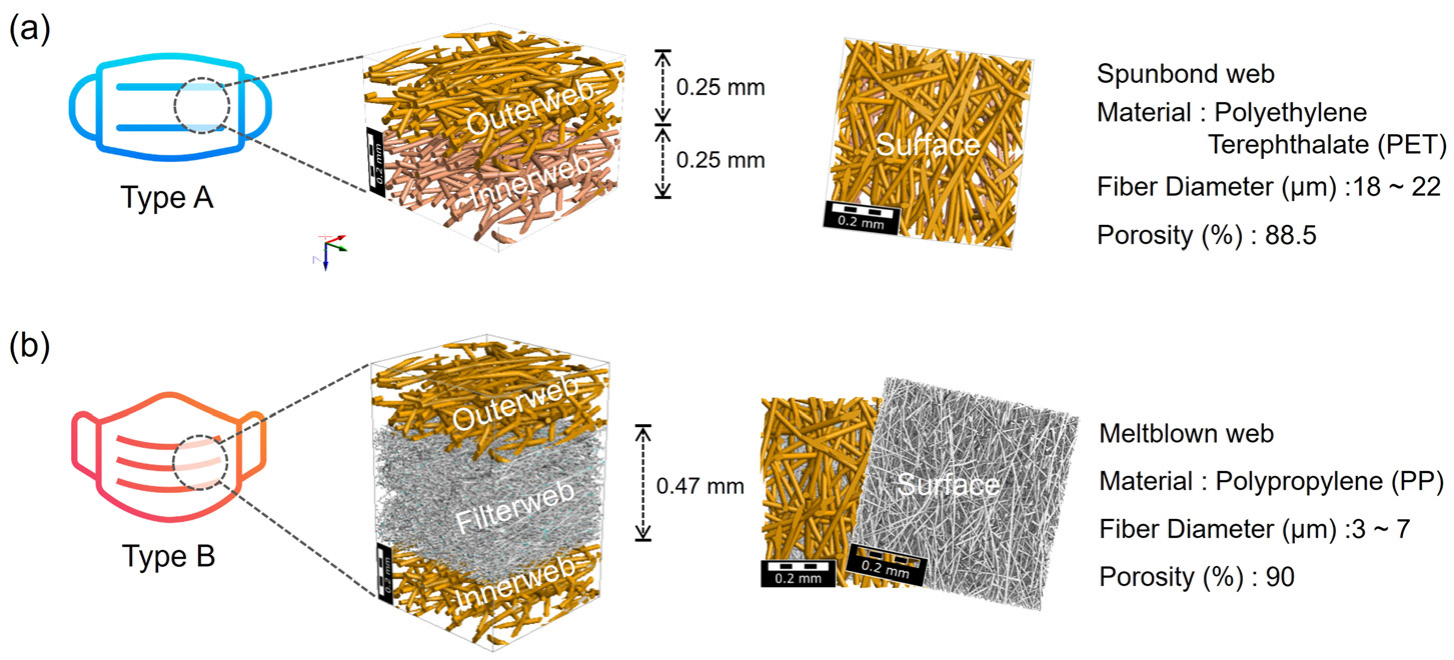
Modeling of face masks: (a) Type A mask with two layers of materials and (b) Type B mask with three layers of materials.

Two types of virtual webs of face masks were constructed using the FiberGeo^®^ module of the GeoDict^®^ program.^28,29^ The reference nonwoven materials constructed for the webs of virtual masks were as follows: a spunbond web with 9.3% ± 0.7% filtration efficiency and a meltblown web with 95.0% ± 0.7% filtration efficiency. The Type A mask was modeled by the construction of two layers of spunbond webs, mimicking an uncertified droplet mask. The Type B mask was modeled with one layer of a meltblown filter web sandwiched with outer and inner spunbond webs, mimicking a KF94-certified face mask. The virtual construction processes of the masks and the constituent material parameters are shown in Figs. 3(a) and 3(b).

**FIG. 4.**
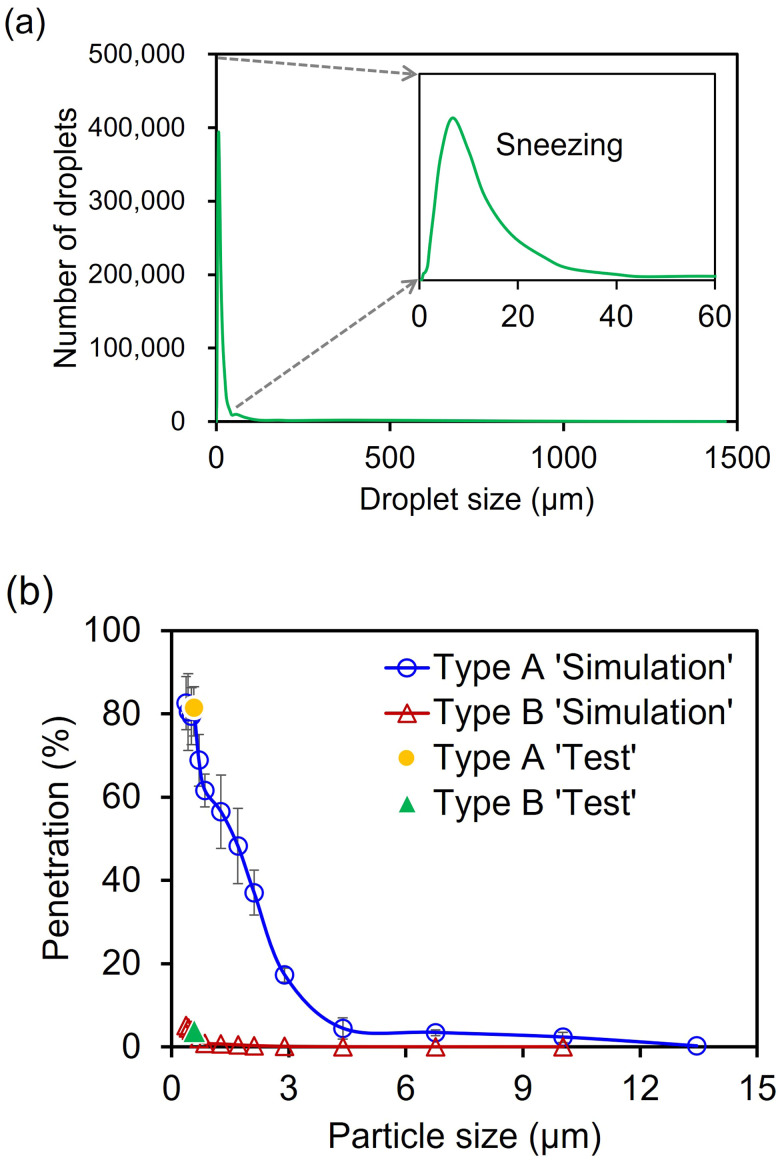
Sneezing droplets characteristics and particle penetration of virtual masks: (a) the size distribution of sneezing droplets referenced in previous studies^31,32^ and (b) fractional NaCl penetration of virtual masks for different particle sizes. Yellow round and green triangular dots are the actual particle penetration results of the corresponding particle size.

**FIG. 5.**
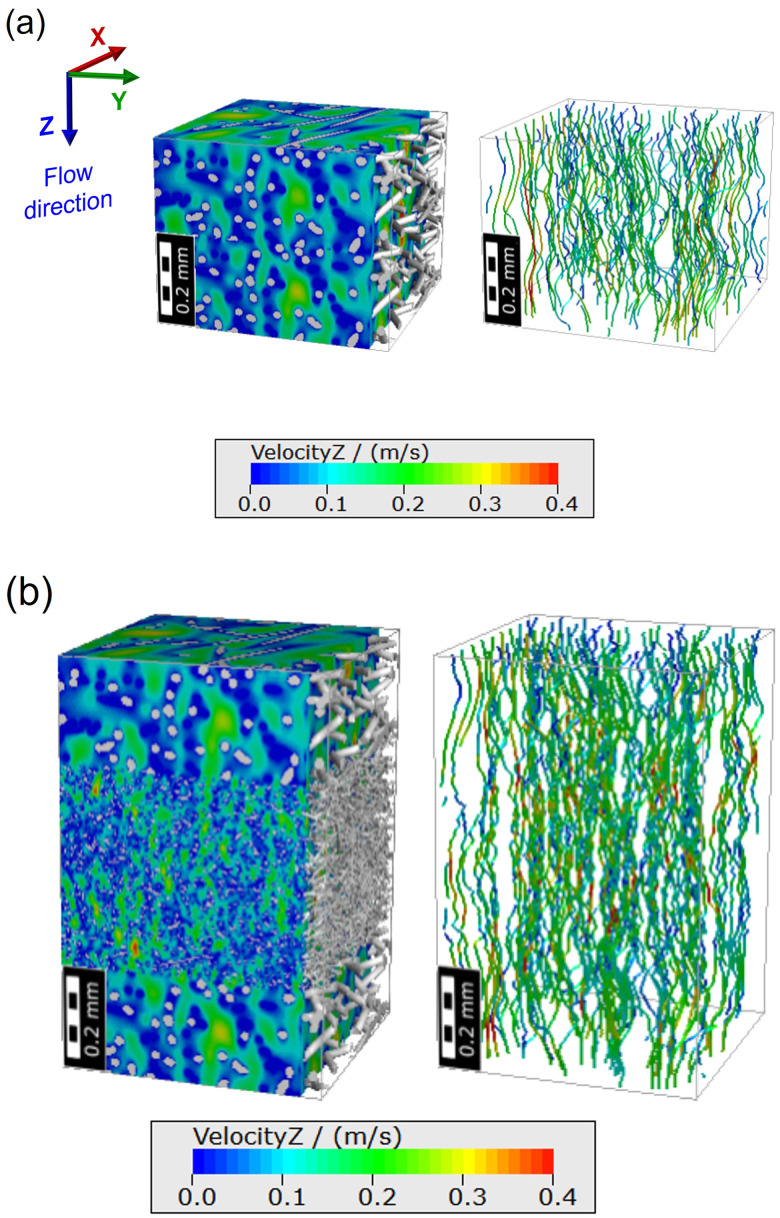
Velocity distribution and velocity streamline in filter media: (a) Type A and (b) Type B.

The droplet size distribution was assumed to be spherical and was set from 0.4 to 1500 *μ*m, referring to previous studies,^31,32^ and the most of the droplets ranged in size from 0.4 to 50 *μ*m [Fig. 4(a)]. Assuming a slow air flow through the filter medium with a small Reynold number (≪1), the balance of momentum equation was simplified to the Stokes equation. The particle penetration percentage (i.e., 100% filter efficiency %) was calculated by the FilterDict^®^ module of GeoDict^®^. In the simulation, the size of the constructed model of the filter webs were (0.625 × 0.625 × 0.5) mm^3^ for Type A and (0.625 × 0.625 × 0.97) mm^3^ for Type B, respectively. For the simulated boundary conditions, the exposed area, particle concentration, and flow rate were set to be the same as the test conditions. A previous study reported that sneezing could introduce as many as 40 000 droplets of which size ranges 0.5 to 12 *μ*m.^31^ In the filtration simulations, the particle was interpreted in the form of a sphere reflecting the density. As the overlapping particles share the volume, overall volume loss occurs. If the volume loss is too high, the accuracy of the analysis decreases, so the number of particles per batch must be reduced. Through this process, the maximum number of particles that can be analyzed was determined. Since the efficiency was 100% for all those with a particle size of 13 *μ*m or more, the number of particles between 0.4 and 13 *μ*m was analyzed as 1482 to 1834. Table I summarizes the parameters used in the filtration simulation. To verify the predicted filter efficiency, particulate penetration through the webs of virtual masks was compared with the actual test results [Fig. 4(b)]. The actual test was conducted with the average size of one droplet particle, i.e., ∼0.6 *μ*m (mass median diameter). The results were found to be well correlated. In this simulation, we considered the slip between flow and fiber surface due to the small fiber size. In the no-slip boundary condition, the pressure drop could be overestimated for the given mass flux. Figures 5(a) and 5(b) show a three-dimensional view of the velocity fields for the Type A and Type B filters. Although the slip flow was considered, high local velocity fields appeared in Type B. Type B consists of three layers, and the middle layer was composed of a dense and thick layer. By the Bernoulli's effect, high local velocity of the flow in the narrower channel formed in the dense middle layer of Type B was observed. The droplet size distribution and the number of particles from the size-dependent penetration percentage were applied to the subsequent aerodynamic dispersion simulation of the droplet.

**TABLE I.** Filtration simulation parameters.

Parameters	Values
Air density, $\rho_{a}$ (kg/ $m^{3}$)	1.204
Air dynamic viscosity, $\mu_{a}$(kg/m·s)	1.834 × 10^–5^
Particle density^a^, $\rho_{p}$ (kg/ $m^{3}$)	2650
Filter thickness, $t_{f}$ (m)	Type A: 0.0005; Type B: 0.000 97
Face velocity, v (m/s)	0.1

^a^ Particle density is the sneezing droplet's density applied in the simulation.

The inherent permeability *k* was then calculated by applying Darcy's law, as indicated as Eq. (1), where *v* is the face velocity (m/s), ΔP is the pressure drop (kg/m·s^2^) from the flow simulation, $t_{f}$ is the thickness of the total nonwoven layers (m), *k* is the permeability (m^2^), and *μ*_a_ is the dynamic viscosity of air (kg/m·s). From this equation, if the permeability is constant, the pressure drop is proportional to the face velocity

$$k=-v\frac{\mu_{a}t_{f}}{\Delta P}.$$ (1)

The permeability of the modeled structures, Type A and Type B, were 1.54 × 10^−10^ and 2.91 × 10^−11^ m^2^, respectively. The 3D constructed mask was regarded as a porous structure and the calculated permeability values computed from the FlowDict^®^ module and Eq. (1) were applied to the mask properties in the aerodynamic dispersion simulation of the droplets. The maximum transmitted distance of a respiratory droplet through the mask was calculated by means of a CFD analysis.

The face velocity in the microscale filtration simulation was set to 0.1 m/s, while it was set to 4.5 m/s in the macroscale aerodynamic dispersion simulation. According to simulations not included in this paper, the filtration simulation verified with a filter test at 0.1 m/s showed a much higher penetration percentage than at 4.5 m/s with a range of droplet sizes from 1 to 5 μm. However, more than 5 μm droplet size showed equivalent penetrations at both 0.1 and 4.5 m/s face velocities. Moreover, inevitable gaps between reality and filtration simulation exist, such as droplet shapes, breakup, and many other uncertainties associated with filter, droplet, air flow, heat, electrical charges, and interactions. Although it should be verified with further studies, the reality could show a higher penetration than the simulation results. Therefore, as a conservative and harsh condition, results from the filtration simulation at 0.1 m/s, which is experimentally verified, were applied to the macroscale aerodynamic dispersion simulation.

### C. Modeling of a face and face masks with different leakage rates

**FIG. 6.**
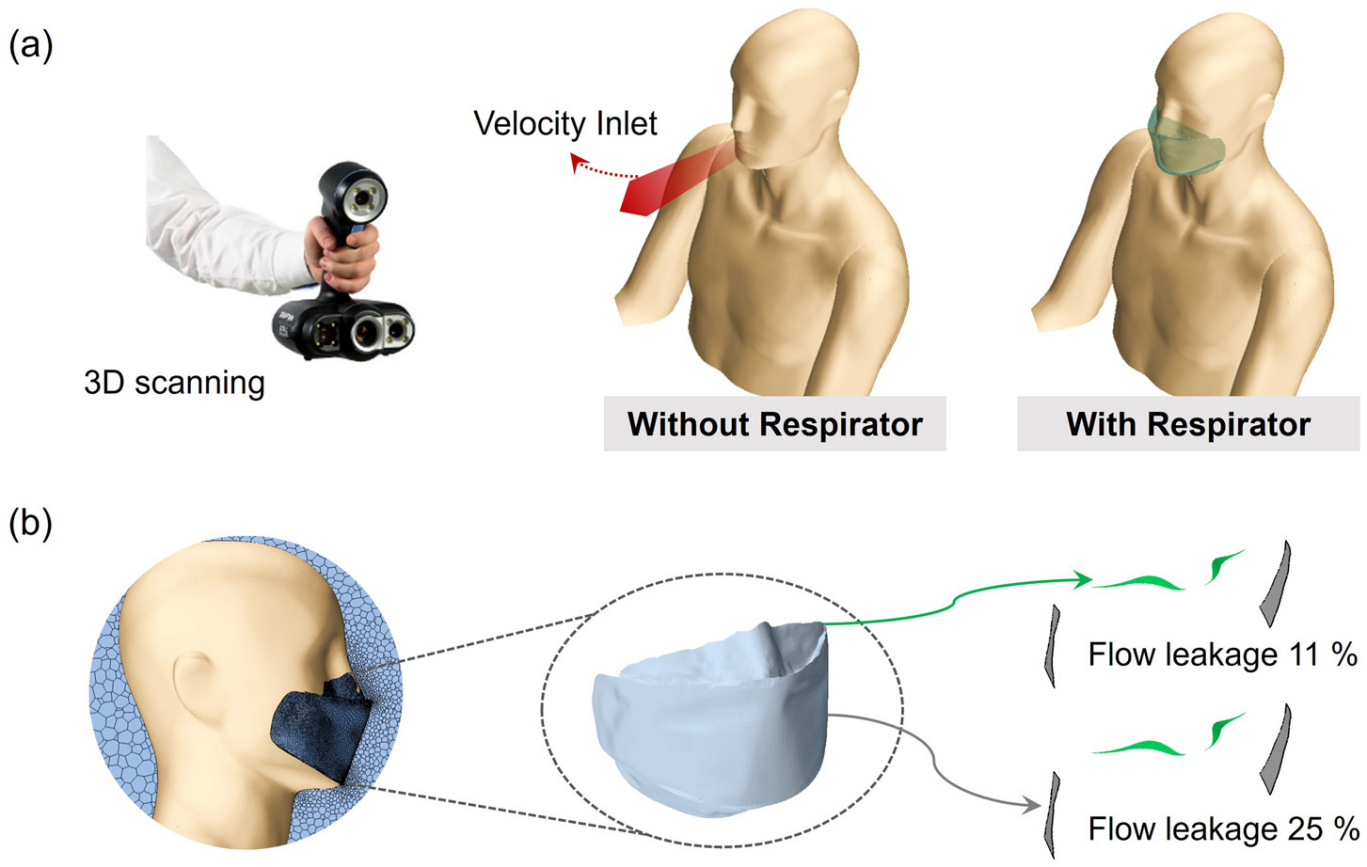
A person's body and a face mask generated via 3D scanning: (a) a person's upper body without (left) and with (right) a face mask; (b) flow leakages of 11% and 25% were set, respectively. Flow leakage only occurs through the periphery of the masks in the nose and cheek regions.

A person's upper body shape and face mask were reverse-engineered by 3D scanning using an optical non-contact scanner (Go!SCAN 50, Creaform Inc., Canada) [Fig. 6(a)].^33,34^ The scanned mesh data with a resolution of 0.5 mm were generated in real time via VXelement software connected to the 3D scanner. The post-treatment software VXmodel, which is integrated into VXelement, enabled the use of the 3D scan data in CAD software. The generated 3D model of the person's body and face mask were utilized in the subsequent simulations.

**FIG. 7.**
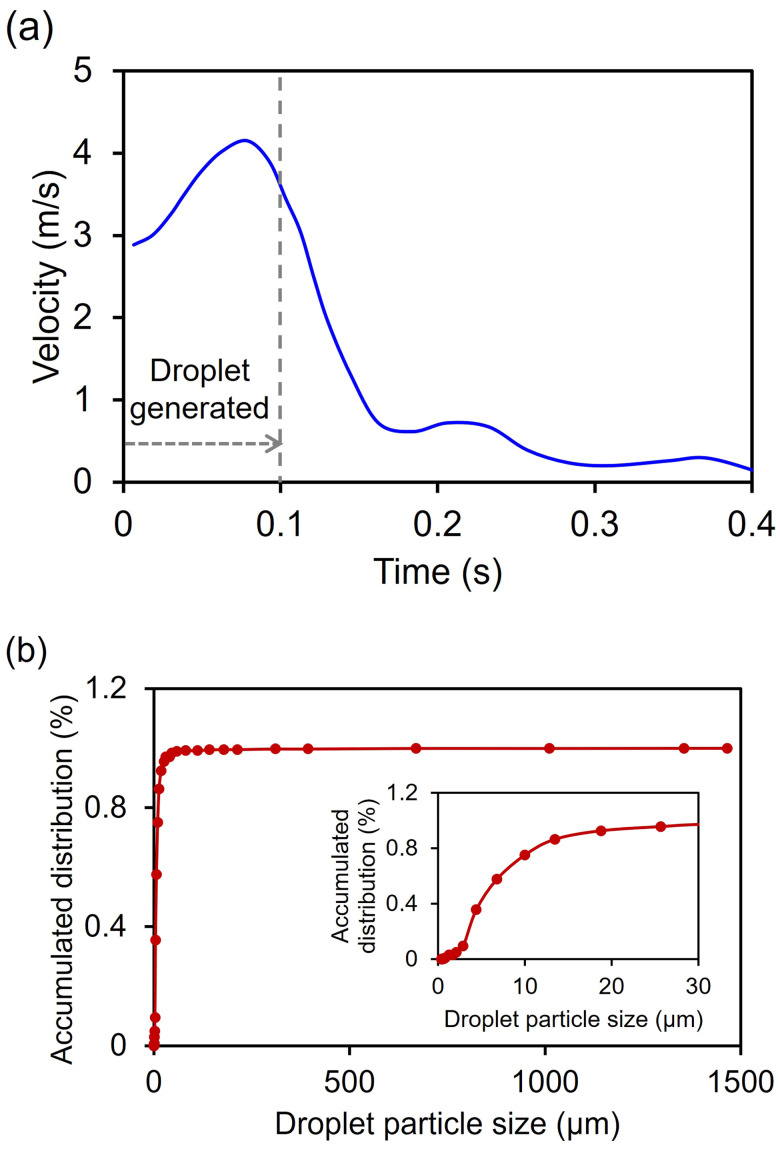
Sneezing droplet modeling: (a) flow velocity profile by sneezing, and (b) accumulated virtual droplet size distribution by sneezing.

**FIG. 8.**
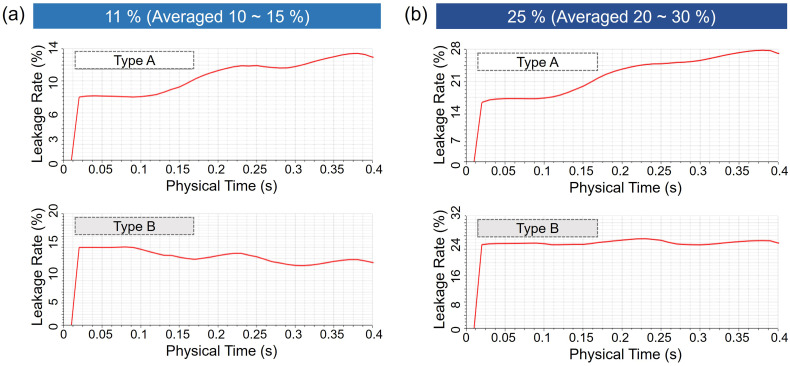
Average leakage rates of face masks for 0.4 s: (a) average leakage rate of 11% for face mask Types A and B, and (b) average leakage rate of 25% for face mask Types A and B.

Face fit is a critical factor that affects the leakage percentage of face masks.^35^ The face fit and the resulting leakage rate are influenced by the 3D shape of a person's face and the face mask.^36,37^ “Leakage rate” (%) was defined by the air flow leakage as a ratio of the flow through an open gap in the nose and cheek regions to the total flow coming out of the mouth, with particle penetration through the mask excluded when calculating the leakage rate [Fig. 6(b)]. From the previous study where the leakage rates of 25 human subjects were investigated, the average leakage rates for a N95 mask and a surgical mask were 3%–5% and 20%–40%, respectively.^38^ From Jung *et al.*'s study, the average leakage rates ranged from 0.0% to 63%, depending on the materials and design of face mask.^39^ In our study, leakage rates of 11% and 25% were applied, corresponding to the leakage criteria of the KF94 and KF80 masks certified by the Korean Food and Drug Administration (KFDA).^40^ While the CFD of this study has limited approach of fixed human position, the leakage rates used as inputs of CFD reflected the realistic average leakage rates set by the regulatory standard. Given that the flow rate generated from the mouth can vary over time, the average leakage rates during 0.4 s were investigated employing the flow velocity profile in Fig. 7(a) [Figs. 8(a) and 8(b)].

### D. Environment configurations and sneezing droplets

**FIG. 9.**
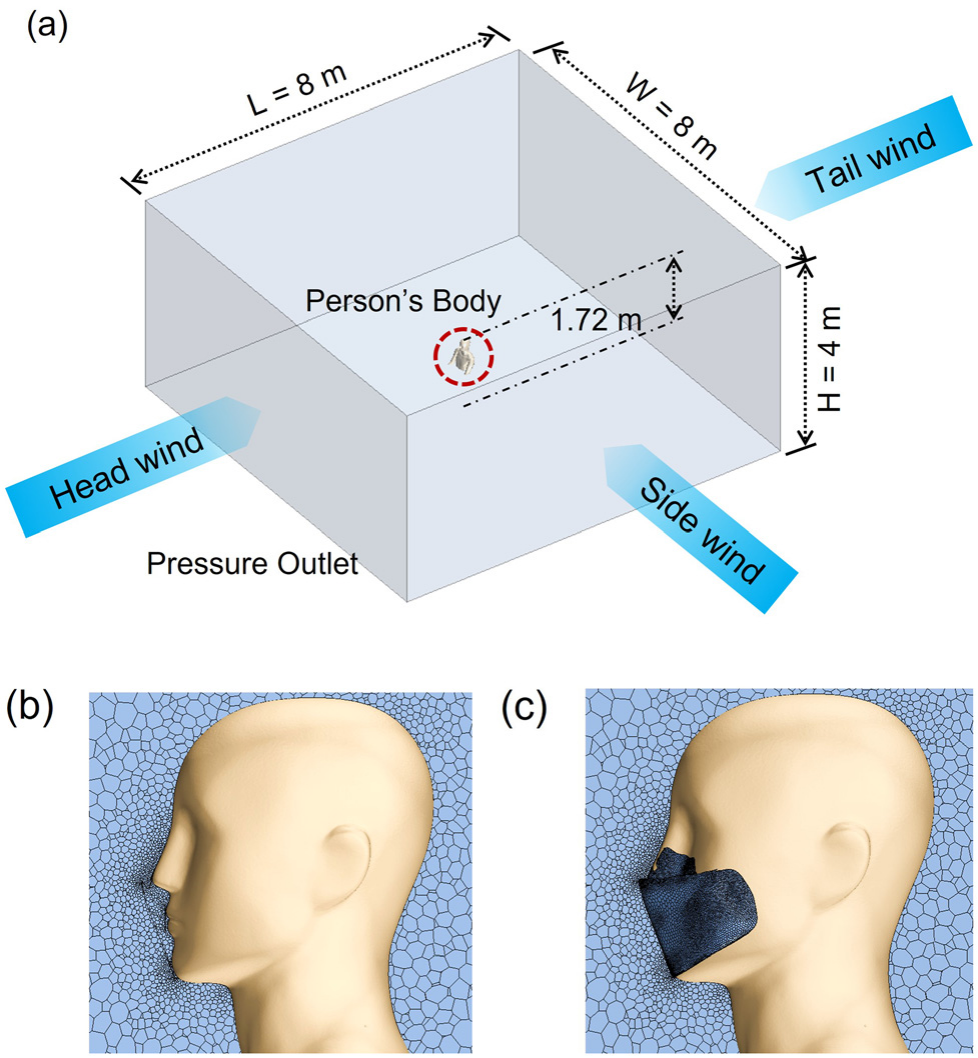
Boundary conditions: (a) Computational domain dimensions and boundary conditions for the indoor environment, and (b) computational grid without a face mask (c) wearing a face mask.

Figure 9(a) shows the computational domain in combination with the dimensions and boundary conditions. A full-scale room for which L × W × H = 8 × 8 × 4 m^3^ was used as an indoor environment for the simulation. This room was ventilated by wind flowing through the walls in front, back, and sides of a person to apply different environmental configurations with three different air flow (wind) directions. The directions of the wind applied were as follows: (1) from front to back (head wind), (2) from back to front (tail wind), and (3) from left to right of the person (side wind). The wind velocity was set to 1.96 m/s, reflecting the average annual wind speed in South Korea in 2019.^41^ The environmental temperature and the relative humidity were set to 20 °C and 70% RH, respectively, considering the average humidity of South Korea.^4,41^ The person's altitude from the floor was designated to be approximately 1.72 m, applying the average height of a man in South Korea as of 2018.^42^ The person's body was set to a fixed position, without any human movement. The person's mouth was defined as the velocity inlet. As the pressure outlet condition, the outermost location of the domain was used. In this study, the optimum number of grids was calculated by performing the grid resolution test, and simulation was performed based on the result. A comprehensive representation of a computational grid for case (1) without and (2) with a face mask was displayed in Figs. 9(b) and 9(c). A grid composed of polyhedral and prism-layer hybrid was generated with unstructured elements of a total number of 2.5 × 10^7^. The grid was well refined the area around the face mask with 3 mm and then gradually coarsened in the field area far from the person with 3 cm intervals. The accuracy of the results highly depends on the grid size, so a dense grid was used near the mouth, especially filtering mask and around the mask. This method helps to save on computational costs by reducing the total number of grids for the complex geometry. The total computational cost, in terms of simulation time, for each case, was three days when using 48 processor cores.

Natural human expiratory flows such as coughing, sneezing, and breathing generate a “jet-like” air flow. Based on previous findings pertaining to the velocity of a sneeze,^43,44^ the maximum velocity of the air flow was set to reach 4.5 m/s within 0.1 s [Fig. 7(a)]. The air flow velocity profile for a period of 0.4 s in Fig. 7(a) was used to simulate the motion of sneezing droplets. The same velocity profile was used for the droplets. The droplet size produced by sneezing was assumed to be mostly less than 50 *μ*m, referring to earlier work.^31,32^ The virtual droplet size distribution generated in the CFD simulation was shown in Fig. 7(b). This was a step to verify whether the droplet size distribution referenced in the literature indicated in Fig. 4(a) is properly implemented. The penetration results of the simulation according to the particle size of the mask in Fig. 4(b) were used for the CFD simulation to calculate the distance of respiratory droplets.

### E. Aerodynamic modeling of respiratory droplets

Three essential governing equations (i.e., continuity equations and balance of momentum and balance of energy equations) are required to describe the dynamics of the droplet mass and energy. They are derived via a nonlinear partial differential equation by referring to the differential control volume. In addition, a turbulent air flow model should be defined to analyze the momentum of the air flow. The governing differential equations are discretized in the physical domain and numerically solved by the finite volume method (FVM), which is well known in computational fluid dynamics (CFD).

#### 1. Continuity equation

The mass conservation principle applied to the control volume at the initial time and elapsed time is expressed as follows:

$$\rho_{0}\left(X\right)=\rho \left(X,t\right)J(X,t),$$ (2)

where $\rho_{0}$ and ρ are the densities at the initial time and the elapsed time, respectively; *X* is the position vector; and *J* is the Jacobian of the volume. Taking the derivative of Eq. (2), one can derive the following continuity equation:

$$\frac{\partial \rho }{\partial t}+\nabla \cdot \left(\rho v\right)=S.$$ (3)

The first term on the left-hand side of Eq. (3) is the transient density change. The second is the divergence of ρv, which is from the Lagrangian convective part of the total derivative of the density. v is the velocity vector. The term (i.e., S) on the right-hand side is the source term. When the source is zero, Eq. (3) implies that the rate of density change at a spatial coordinate is equal to the sum of the density change rate in all directions.

#### 2. Balance of momentum

The balance of momentum describes the conservation of momentum in a differential control volume. In an inertia coordinate system, the balance of momentum equation is expressed as

$$\frac{\partial (\rho v)}{\partial t}+\nabla \cdot \left(\rho \mathrm{vv}\right)=-\nabla p+\rho g+\nabla \cdot \left(\tau +\tau^{t}\right)+F_{\mathrm{int}}+S^{v},$$ (4)

where p is the pressure applied to all phases; g is the gravitational acceleration vector; τ and $\tau^{t}$ are the molecular and turbulent stresses, respectively; $F_{\mathrm{int}}$ is the internal force, and $S^{v}$ is the momentum source. The left-hand-side terms in Eq. (4) are the transient term and the convection term from the left-most side. The right-hand-side terms in Eq. (4) are the static pressure term, the gravitational body force term, the stress tensor term, the internal body force term, and the source term in that order. The balance of momentum equation originates from Newton's second law, which dictates conservation of the internal and external momentum within the control volume. The internal momentum is associated with the expansion, rotation, and deformation of the internal air flow and is expressed in terms of the internal energy and stress. The number of unknown solution variables included in the stresses increases, resulting in non-closure. Therefore, it should be supplemented with turbulence models to introduce closure.

#### 3. Balance of energy

The balance of energy equation is from the first law of thermodynamics, dictating the total energy conservation within the control volume. It is expressed as follows:

$$\frac{\partial (\rho E)}{\partial t}+\nabla \cdot \left(\rho Ev\right)+\nabla \cdot \left(pv\right)=\nabla \cdot \left(k_{\mathrm{eff}}\nabla T\right)+\nabla \cdot \left[\tau \cdot v\right]+f\cdot v+S_{h},$$ (5)

where E is the total energy, $\tau_{v}$ is the viscous stress tensor, $k_{\mathrm{eff}}$ is the effective thermal conductivity, f is the body force, and $S_{h}$ is the source of total energy. The balance of energy equation describes thermal transferring phenomena in a form of convection and diffusion. In addition, it considers energy dissipation caused by the viscosity and thermal transfer by fluid stresses and body forces.

#### 4. Turbulence flow model (realizable k−ε model)

In the turbulence flow model for air, the velocity is decomposed into the average velocity ( ${\bar{v\;}}_{i}$) and the fluctuating velocity component ( $v_{i}^{\prime }$). This process is known as Reynolds averaging,

$$v_{i}={\bar{v\;}}_{i}+v_{i}^{\prime }.$$ (6)

Substituting Eq. (6) into the balance equations, we can obtain the Navier–Stokes equation in terms of the average velocity and fluctuating velocity, referred to as the compressible Reynolds-averaged Navier–Stokes equation (RANS). In this paper, the realizable k−ε model is selected.

### F. A fully coupled Eulerian–Lagrangian multiphase model

In the Eulerian–Lagrangian multiphase model, the continuous fluid phase is described by the Eulerian method, and the droplet phase is tracked by the Lagrangian method. Lagrangian tracking of particles is performed by integrating the particle path in the discretized physical domain from the corresponding injection point until the integration constraints are satisfied. The momentum of droplets is conserved following Newton's second law:

$$m_{p}\frac{du_{p}}{\mathrm{dt}}=F_{B}+F_{D}+F_{M}+F_{P}.$$ (7)

Here, $F_{B}$, $F_{D},\;F_{M}$, and $F_{P}$ are the gravitational body force, Stokes drag, added-mass forces, and pressure force, respectively, and $u_{p}$ is the velocity of the droplet.^45^ The velocity differences of the air and the droplet are included in $F_{D}$ and $F_{M}$, coupling the CFD airflow with discrete droplets. In addition to the velocity of the droplets, the mass and temperature dynamics of the droplets are considered in this paper. The coupled models used here for the heat and mass of respiratory droplets are explained below.

#### 1. Stokes drag model of droplet-airflow interaction

$F_{D}$, the Stokes drag, is defined as

$$F_{D}=\frac{1}{2}C_{D}A_{p}\left|u-u_{p}\right|\left(u-u_{p}\right),$$ (8)

where $C_{D}$ is the drag coefficient of the droplet and $A_{p}$ is the projected area of the droplet. The drag coefficient is a function of the small-scale flow features around individual droplets. These features are impractical to resolve spatially. Therefore, the usual practice is to obtain the drag coefficient from correlations derived from an experiment or theoretical studies. These correlations depend on the nature of the droplet.

The Schiller–Naumann correlation^46^ or the drag coefficient is applied as follows:

$$C_{D}=\left\{\begin{array}{ll} \frac{24}{{\text{Re}}_{p}}\left(1+0.15{\text{Re}}_{p}^{0.687}\right), & {\text{Re}}_{p}\leq 10^{3},\\ 0.44, & {\text{Re}}_{p}>10^{3}, \end{array}\right.$$ (9)

where ${\text{Re}}_{p}$ is the droplet Reynolds number.

#### 2. Droplet evaporation and heat transfer

Mass in a fluid is transferred by absorption and evaporation. Mass changes of a saliva droplet by vaporization are driven by water transport from the droplet surface to the surrounding ambient airfield. Assuming that an exhaled droplet evaporates in a quiescent condition at a fixed temperature and relative humidity, the evaporation rate is expressed as^47^

$${\dot{m}}_{p}=-g^{*}A_{s}ln\left(1+B\right),$$ (10)

where B is the Spalding mass transfer number and $A_{s}$ is the surface area of the droplet. *B* is widely used for analyzing the surface area vaporization process of droplets under low pressure conditions, as

$$B=\frac{Y_{v,s}-Y_{v}}{1-Y_{v,s}},$$ (11)

where $Y_{v,s}$ is the mass fraction of water vapor in the air near the surface of the droplet and $Y_{v}$ is the mass fraction far away from the droplet surface. $g^{*}$ in Eq. (10) is the mass transfer conductance, which depends on the mass fraction dynamic diffusivity and Sherwood number

$$g^{*}=\frac{\rho D_{v}Sh_{p}}{D_{p}}.$$ (12)

$D_{v}$ is the mass diffusivity of the water vapor, ${Sh}_{p}$ is the Sherwood number, and $D_{p}$ is the mass diffusivity of the liquid droplet. The Sherwood number defines the non-dimensional ratio of the convective mass transfer to the diffusion mass transfer. Generally, the Ranz–Marshall correlation model is applied, as shown below^48,49^

$$Sh_{p}=2\left(1+0.3Re_{p}^{\frac{1}{2}}Sc^{\frac{1}{3}}\right).$$ (13)

In this equation, ${Re}_{p}$ is the Reynolds number of the droplet and Sc is the Schmidt number. The Schmidt number defines the ratio between the viscous and mass diffusion rates.

The temperature evolution of the droplet is solved by the enthalpy equation (14), as

$$\frac{de_{p}}{\mathrm{dt}}=m_{p}c_{p}\frac{dT_{p}}{\mathrm{dt}},$$ (14)

where $e_{p}$ is the enthalpy difference that evolves over time, $c_{p}$ is the specific heat capacity of the droplet and $T_{p}$ is the droplet temperature. While the temperature evolution of the droplet was computed, the result was negligible at < 1 °C. In the model, the secondary breakup of droplets was not considered. All computational simulations were conducted by STAR-CCM+, which is an industrial-grade multiphysics simulation software program.

## III. RESULTS AND DISCUSSION

In the simulation in this study, the droplet propagation characteristics were predicted under the following conditions: (1) wearing face masks with different filtration efficiency rates, (2) wearing face masks with different leakage rates, and (3) wearing face masks in a still or windy environment, where the wind flow directions were varied. The various factors included in the simulation study are shown in Table II. Case 1 is the simulation without a face mask, and Cases 2–8 are simulations with a mask with various performance capabilities in different ambient flow conditions.

**TABLE II.** Various mask-wearing situations included in the simulation.

Case study	Material	Component	System
Case 1	Without filtering mask	⋯	Base
Case 2	Type A	11% leakage Rate	Base
Case 3	Type A	25% leakage Rate	Base
Case 4	Type B	11% leakage Rate	Base
Case 5	Type B	25% leakage Rate	Base
Case 6	Type B	11% leakage Rate	Head wind
Case 7	Type B	11% leakage Rate	Tail wind
Case 8	Type B	11% leakage Rate	Side wind

**FIG. 10.**
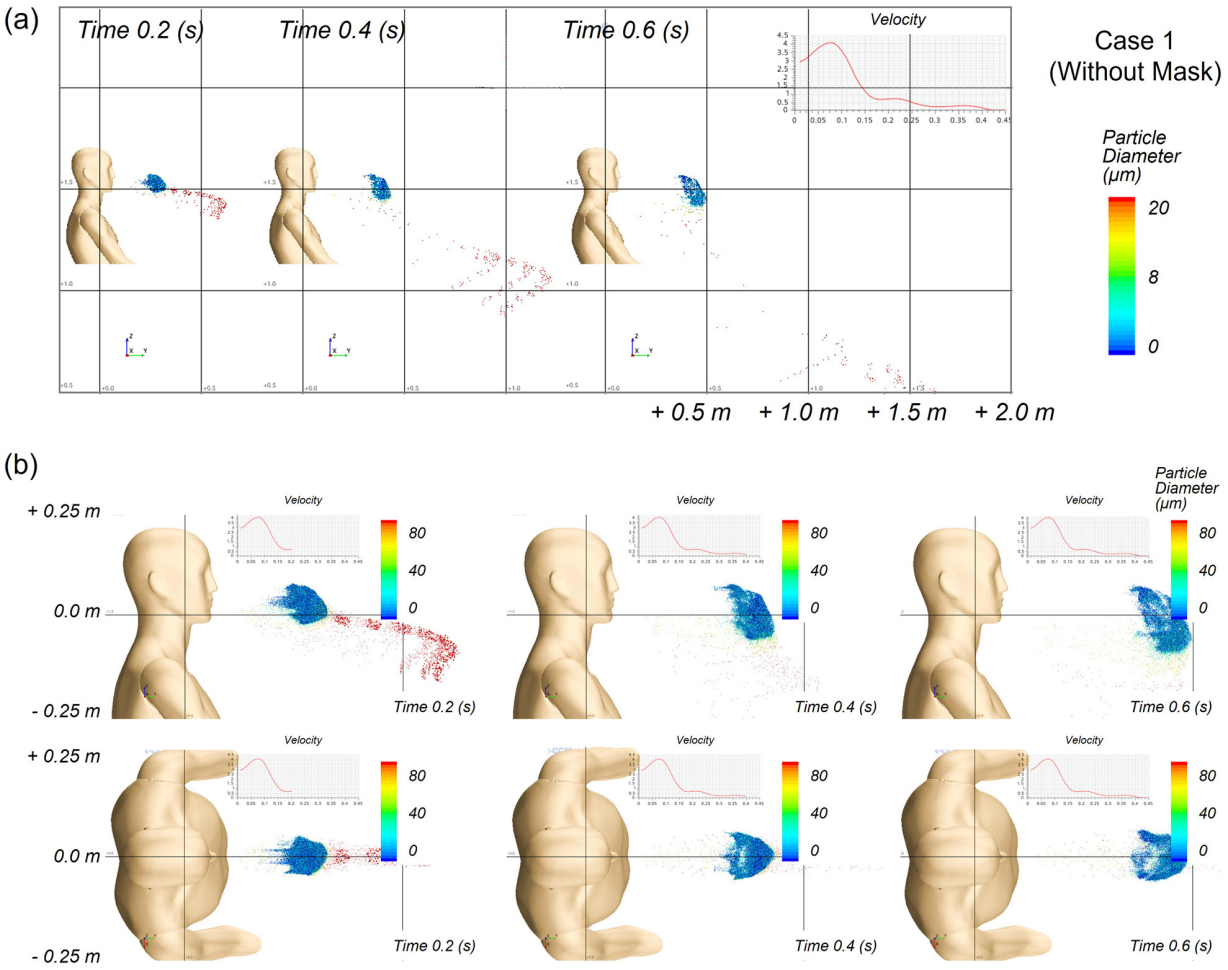
Transmitted distance of sneezing droplets over time without a face mask, simulated for Case 1: (a) side view, (b) enlarged side view and top view for droplet movement.

The maximum distance of droplet propagation in the forward and side directions, without a mask, was investigated as Case 1. Droplet movement from 0.2 to 0.6 s is shown in Figs. 10(a) and 10(b), where larger droplets are denoted by the red dots and smaller droplets are shown in blue. Relatively large droplets of 1000–1300 *μ*m moved up to 2 m forward and 0.12 m in the side directions. When the evaporation of droplets was considered in the simulation, droplets of relatively small size evaporated and disappeared before they reached the floor. Thus, small liquid droplets could not move to a long distance.

**FIG. 11.**
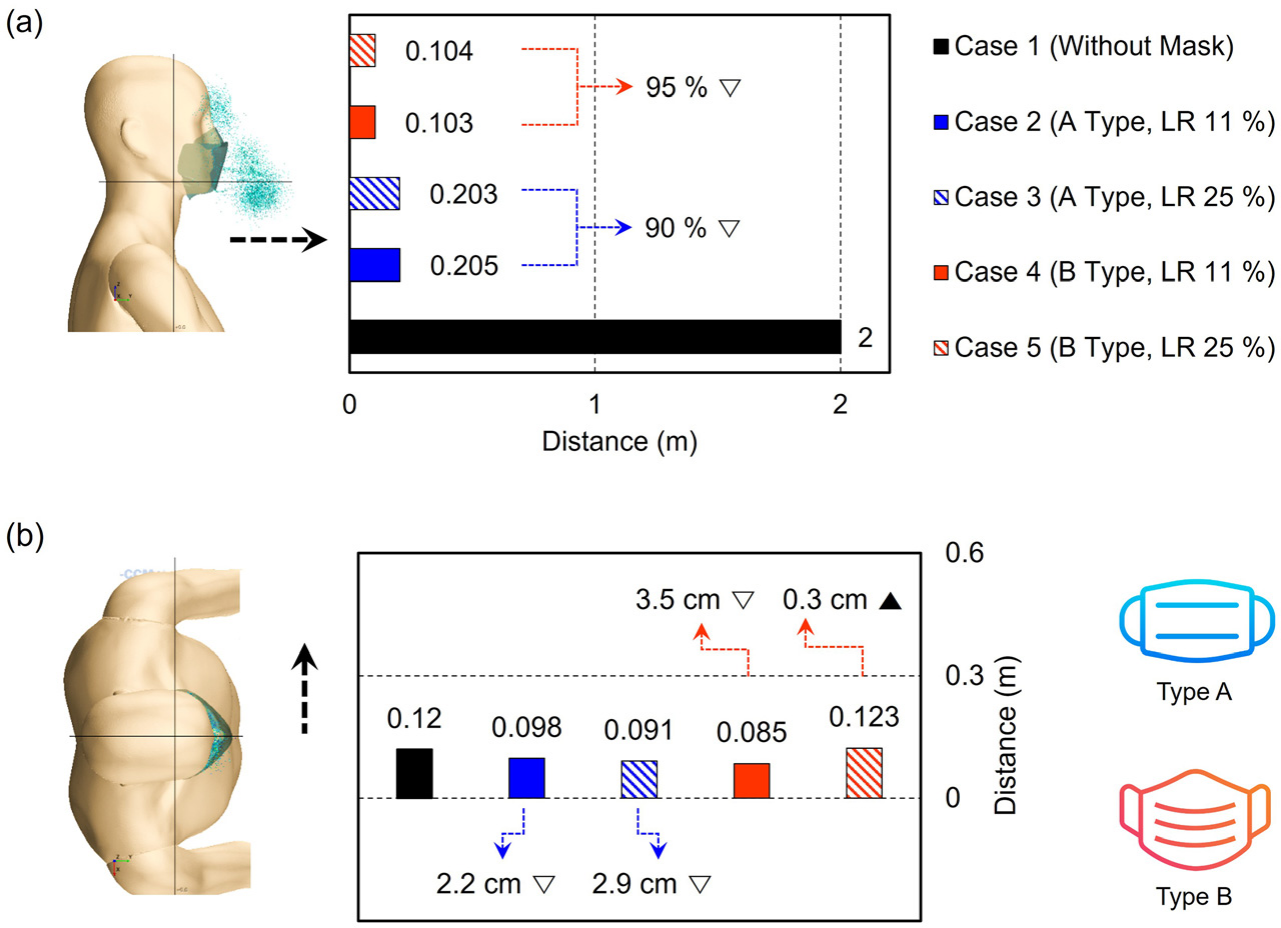
Distance transmitted to the front and side directions of droplets for different mask types with different leakage rates (abbreviated as LR): (a) in the front direction and (b) in the side direction.

With any type of mask wearing, the forward transmittance distance of droplets was significantly decreased (90%–95% reduction of distance) compared to Case 1 (no mask). The lateral transmittance was barely affected by wearing any type of mask. Droplet transmittance when wearing a Type A mask was simulated as Case 2 and Case 3, with leakage rates of 11% and 25%, respectively. Compared to Case 1 without a mask, the movement of droplets with a face mask was considerably restricted in the forward direction, showing a reduction of approximately 90% of the transmittance distance (transmittance distance of 0.203–0.205 m) [Fig. 11(a)]. Notably, there was little difference in the transmittance distance regardless of the leakage rates of the masks, showing that droplets in the forward direction are mostly droplets that penetrated through the mask material and not those that leaked through the nose and cheek regions. Between mask types, Type B (∼96.1% filtration efficiency) showed a half the transmittance distance of Type A (∼18.5% filtration efficiency). From Fig. 4(b), which shows the simulated fractional penetration with various droplet sizes, both the spunbond and meltblown materials showed filtration efficiency rates that exceeded 90% when the particle size was greater than 4.5 *μ*m. These results demonstrate that by preventing large particles from being transmitted through the mask, the overall droplet transmittance distance can be reduced.

**FIG. 12.**
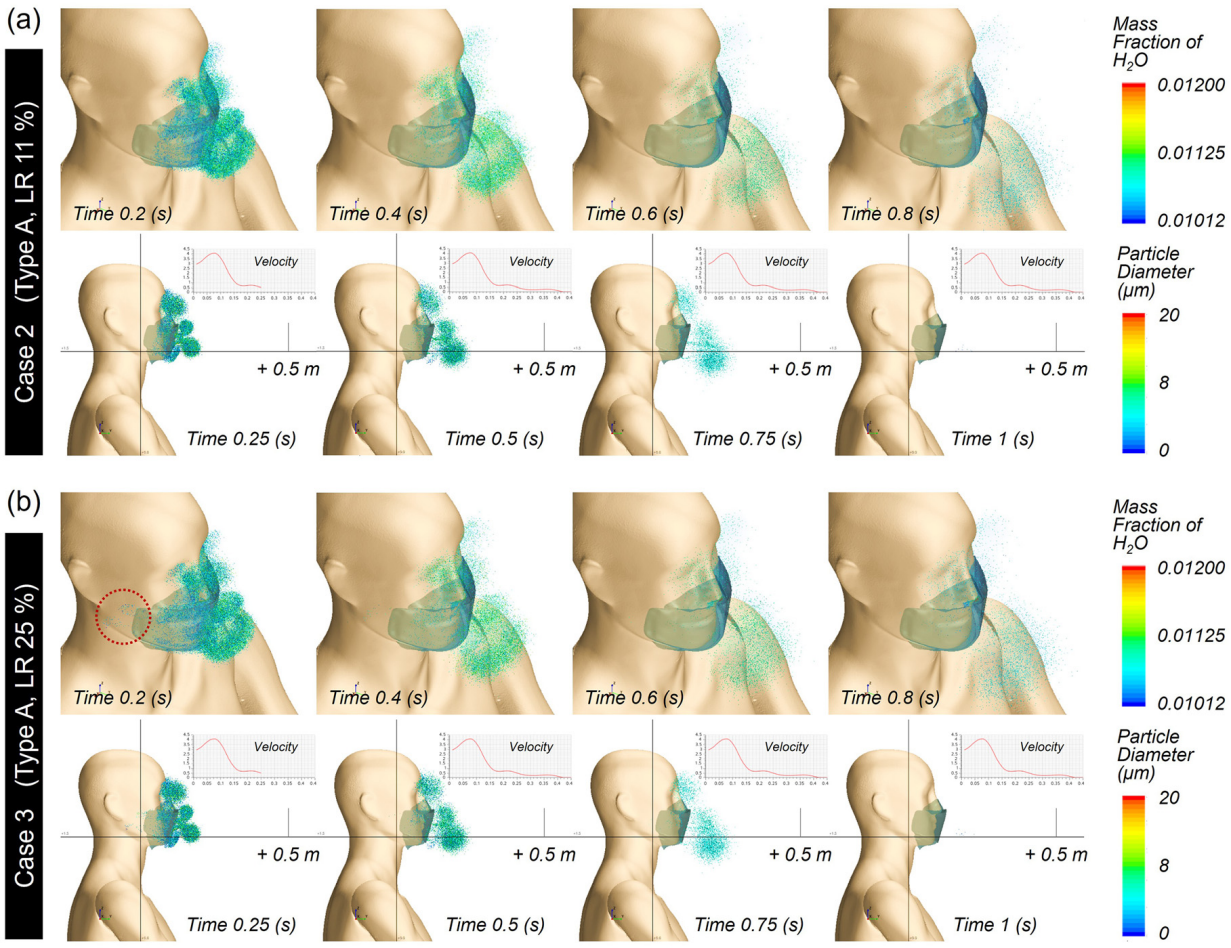
Droplet trajectory profile of mask type A: (a) Case 2, (b) Case 3. “Mass Fraction of H_2_O” is the mass fraction of water vapor in air when the droplet evaporates, and a larger value means a higher mass of droplet evaporation.

Unlike the forward direction, the lateral transmittance of droplets was considerably limited even without mask wearing, as the droplets were ejected mostly in the forward direction [Fig. 11(b)]. The differences in the transmittance distance among the cases were negligible, indicating that the leakage rate or filtration efficiency of the face mask did not significantly affect the lateral transmittance of droplets when sneezing. The trajectory profile of the droplets was traced by time, as shown in Figs. 12(a) and 12(b) for Case 2 and Case 3 from different view angles. In addition to the droplets transmitted forward through the mask, upward movement of droplets through the nose gap was observed in both cases. These droplets were densely distributed in front of the face and eye area during the first 0.2 s and mostly disappeared from the facial area 1 s after sneezing.

**FIG. 13.**
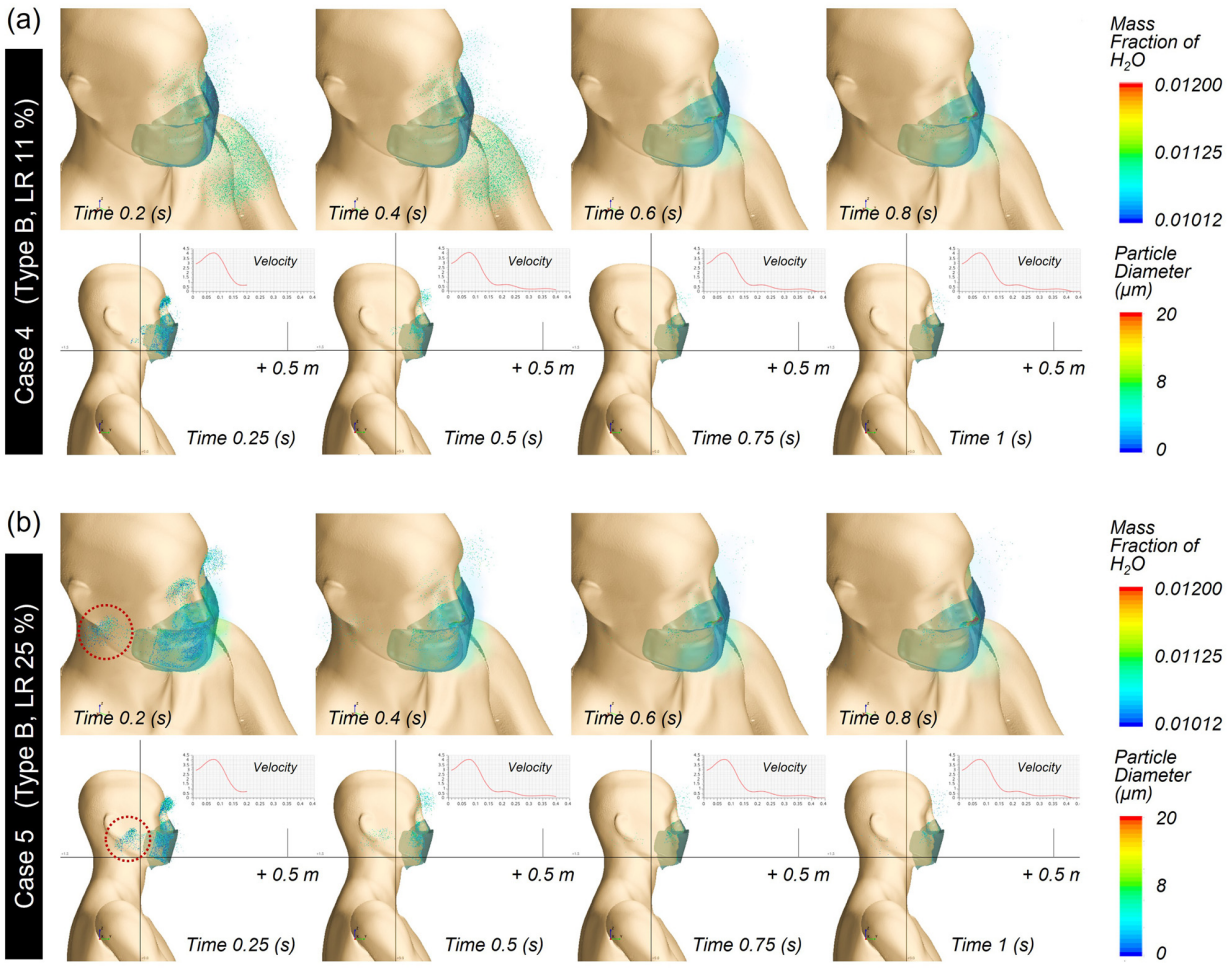
Droplet trajectory profile of mask type B: (a) Case 4 and (b) Case 5.

For Case 4 and Case 5 with a high-efficiency face mask, the droplet cloud around the facile area decreased noticeably compared to those in Case 2 and Case 3 [Figs. 13(a) and 13(b)], and the droplets moved a shorter distance.

These results clearly demonstrate that the addition of meltblown filter media in the Type B mask was indeed effective as a preventive measure, hindering the spreading of droplets from the mask wearer to others. Overall, the aerosol transmittance distance either in front or on the side was scarcely affected by the leakage rates. While not being significant, a very marginal increase in the transmittance distance in the lateral direction was observed with 25% leakage. This likely occurred because with the high-efficiency filter layer, sneezing would create a relatively high differential pressure between the inside and outside of the mask, which may in turn promote droplet ejection through the open space around the cheek. Overall, mask wearing effectively restricted the moving distance of large droplets regardless of the mask type.

**FIG. 14.**
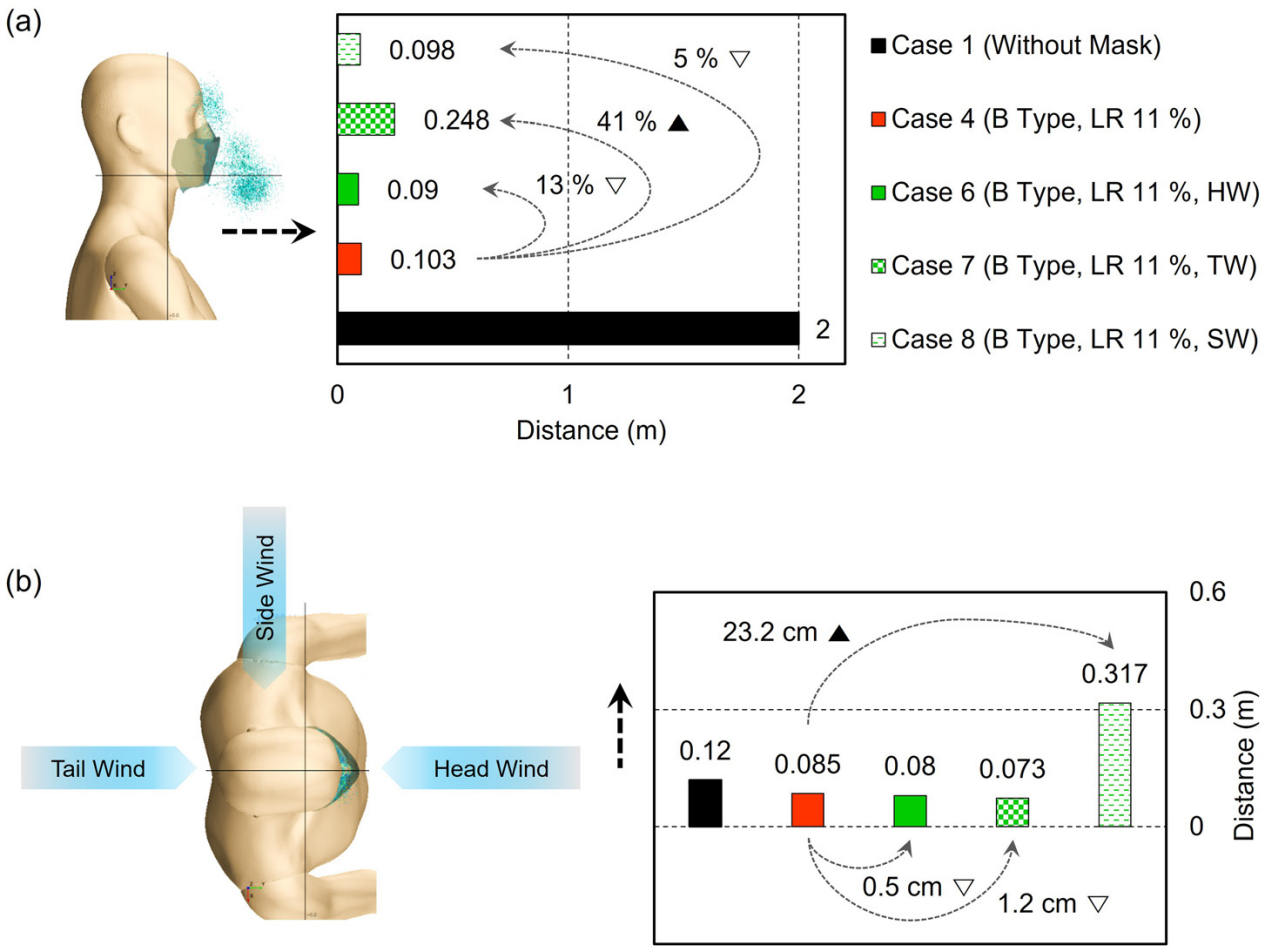
Distance transmitted to the front and side directions of droplets in a windy environment: (a) in the front direction and (b) in the side direction (left to right).

**FIG. 15.**
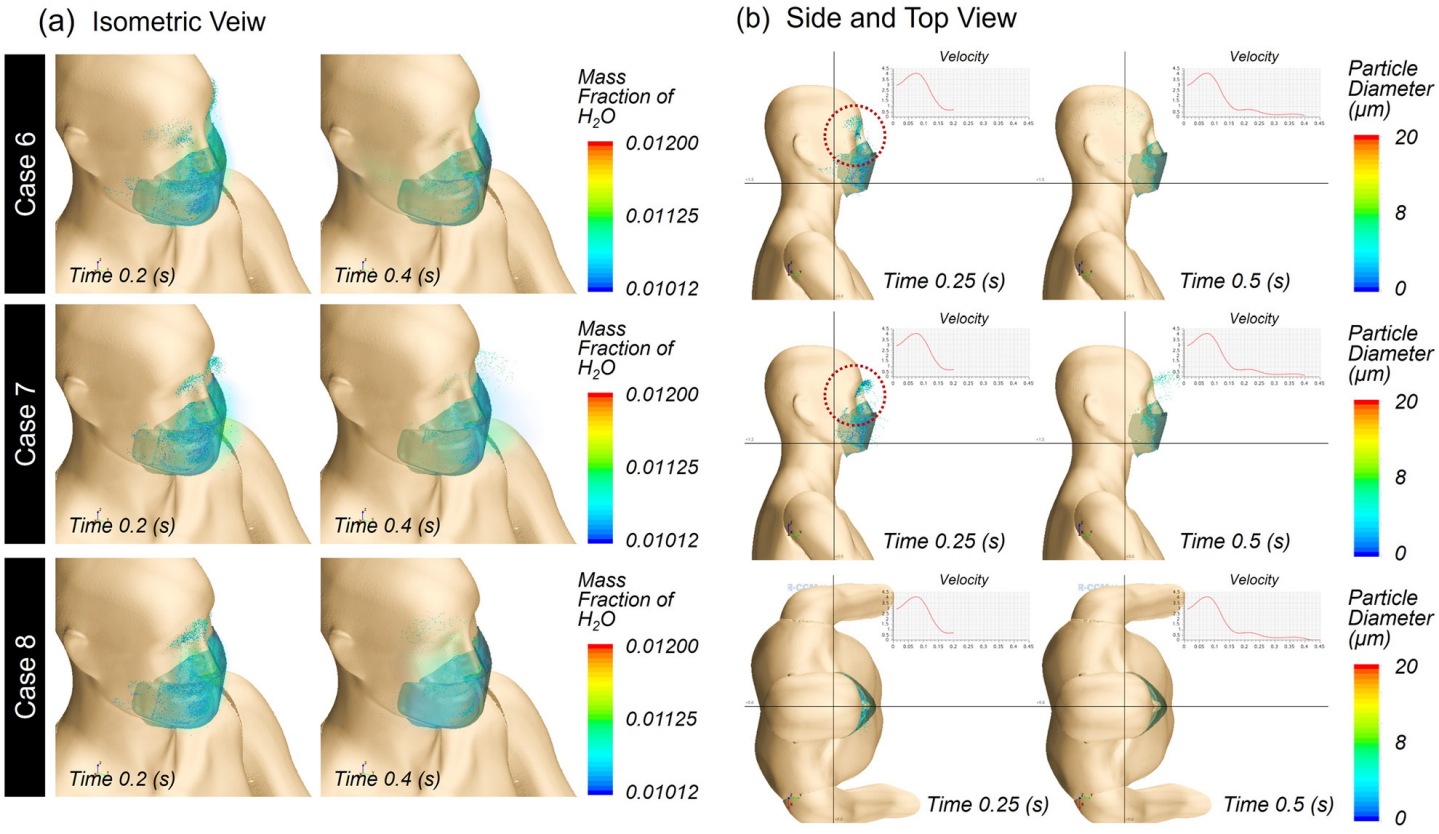
Droplet behavior from 0 to 0.5 s: (a) isometric view in Case “6”–Case “8” and (b) side view in Case “6”–Case “7,” and top view in Case “8.”

The effect of the wind flow on the droplet transmittance was investigated using the Type B mask with 11% leakage. To mimic wind in different directions, head wind (front to back, Case 6), tail wind (back to head, Case 7), and side wind (left to right of face, Case 8) types were applied at a speed of 1.96 m/s in the simulations [Figs. 14(a) and 14(b)]. Compared to Case 4 without wind, droplet movement in Case 6 (head wind) or Case 8 (side wind) was slightly reduced in the forward direction (Case 4, 10.3 cm to Case 6, 9.0 cm), as the wind coming toward the face or side somewhat offset droplet movement in the forward direction. With tail wind, the droplet moved further by 41% from the Case 4, up to 24.8 cm. The trajectory profile of the droplets was traced by time, as shown in Figs. 15(a) and 15(b) for Case 6, Case 7, and Case 8 from different view angles. These results demonstrate that the effect of the wind direction was greater than that of the mask material (Type A or Type B) on the moving distance of the droplets, as large droplets were those that traveled further (Fig. 10), with these particles mostly filtered by the tested mask configurations [Fig. 7(b)]. These findings show that wearing a mask and a relevant social distance depending on the wind condition are necessary to protect against the spreading of contagious droplets to nearby people.

## IV. CONCLUSION

The fully coupled multiphase fluid dynamics associated with droplet propagation by sneezing was investigated by simulating the transmittance of droplets through various mask microstructures under different ambient flow conditions. Spunbond and meltblown materials of the types commonly applied to face masks were modeled to generate two different face mask types with various filtration efficiency rates. The leakage rates of masks were controlled at 11% and 25% through the open gap in the nose and the cheek area. To determine the propagation of droplets, a particle penetration profile with various droplet sizes was numerically determined considering complex air flows and flow-particle interaction within heterogeneous filter media. The statistical droplet size distribution and filter permeability from microscale filtration simulations were linked to a macroscale droplet dispersion simulation.

The simulation results showed that wearing any mask type included in this study can effectively reduce the droplet transmittance distance when sneezing. When not wearing a mask, the maximum droplet propagation distance in the forward direction was 2 m, where larger droplets traveled further. As larger droplets were filtered by either a spunbond or meltblown web structure, the transmittance distance of droplets in the forward direction was significantly reduced to approximately 20–25 cm when wearing a face mask. Between mask types, a KF94 mask with higher filtration efficiency showed a half droplet transmittance distance than a droplet mask. The effects of the leakage rates on the transmittance distance were negligible, as the open gaps existing in the nose and cheek areas scarcely affected the forward or lateral transmittance of droplets. The wind flow direction affected the transmittance distance; droplets affected by a tail wind moved 41% further in the forward direction than in still environment, and it is recommended to keep at least 25 cm distance between people.

This study is significant in that droplet transmittance was investigated by simulating realistic situations and considering various parameters, including the porous filter microstructure, the leakage rates of masks, and ambient flow conditions. This study considered only the sneezing condition as one type of harsh spreading situation. Simulations of various human activities, such as talking and coughing, also should be explored further to predict more precisely the spread of aerosols and droplets. This study proposes a new multiscale and multiphysics simulation methodology for designing mask filter materials.

## AUTHOR CONTRIBUTIONS'

The manuscript was written through contributions of all authors. All authors have given approval to the final version of the manuscript.

## Data Availability

The data that support the findings of this study are available from the corresponding authors upon reasonable request.
